# COMPARISON BETWEEN INGUINAL HERNIOTOMIES WITH AND WITHOUT INCISING EXTERNAL OBLIQUE APONEUROSIS: A RANDOMIZED CLINICAL TRIAL

**DOI:** 10.1590/0102-6720201700030006

**Published:** 2017

**Authors:** Shahnam ASKARPOUR, Mehran PEYVASTEH, Shaghayegh SHERAFATMAND

**Affiliations:** 1 Ahvaz Jundishapur University of Medical Sciences, Department of Pediatric Surgery, Imam Khomeini Hospital, Ahvaz, Khouzestan, Iran

**Keywords:** Hernia, inguinal, Inguinal canal, Hernia

## Abstract

**Background:**

Inguinal herniotomy is the most common surgery performed by pediatric surgeons.

**Aim:**

To compare the results and complications between two conventional methods of pediatric inguinal herniotomy with and without incising external oblique aponeurosis in terms of recurrence of hernia and other complications.

**Methods:**

This one blinded clinical trial study was conducted on 800 patients with indirect inguinal hernia. Inclusion criterion was children with inguinal hernia. The first group underwent herniotomy without incising external oblique aponeurosis and second group herniotomy with incising external oblique aponeurosis. Recurrence of hernia and other complications including ileoinguinal nerve damage, hematoma, testicular atrophy, hydrocele, ischemic orchitis, and testicular ascent were evaluated.

**Results:**

Recurrence and other complications with or without incising external oblique aponeurosis had no significant difference, exception made to hydrocele significantly differed between the two groups, higher in the incision group.

**Conclusion:**

Herniotomy without incising oblique aponeurosis can be appropriate choice and better than herniotomy with incising oblique aponeurosis. Children with inguinal herniotomy can be benefit without incising oblique aponeurosis, instead of more interventional traditional method.

## RESUMO

**Racional:**

Herniotomia inguinal é a operação mais comum realizada por cirurgiões pediátricos.

**Objetivo:**

Comparar os resultados e complicações entre dois métodos convencionais de herniotomia inguinal pediátrica, com e sem incisão de aponeurose oblíqua externa, em termos de recorrência de hérnia e outras complicações.

**Métodos:**

Este estudo cego foi realizado em 800 pacientes com hérnia inguinal indireta. Os critérios de inclusão foram crianças com hérnia inguinal. O primeiro grupo foi submetido à herniotomia sem incisão de aponeurose oblíqua externa e o segundo grupo herniotomia com ela. Foram avaliadas recorrência da hérnia e outras complicações, incluindo lesão do nervo ileoinguinal, hematoma, atrofia testicular, hidrocele, orquite isquêmica e ascensão testicular.

**Resultados:**

A recorrência e outras complicações com ou sem incisão da aponeurose oblíqua externa não apresentaram diferença significativa, com exceção feita à hidrocele significativamente diferenciada entre os dois grupos, maior no grupo com incisão.

**Conclusão:**

A herniotomia sem incisão da aponeurose do oblíquo externo pode ser escolha adequada e melhor do que a herniotomia com incisão dela. As crianças com herniotomia inguinal podem ser beneficiadas sem incisão da aponeurose, em vez do método tradicional mais intervencionista.

## INTRODUCTION

Repair of inguinal hernia in children is the most common and main pediatric surgical modern procedure^6^. It requires closing the opened vaginalis processus, in other words, herniotomy. This type of hernia in a child is considered indication for surgery. Hernioplasty in adults requires the inguinal canal reconstruction and, due to this reason, it is different from pediatric hernioplasty. Inguinal hernia in men is more common than in women and, in men, occurs more often on the right side than the left. In infants due to inguinal hernia ring tight, there is a high risk of hernia incarceration^4^. Elective pediatric inguinal hernia repair stages are different between surgeons. But all of them believe that the main point of surgery is based on accurate anatomy understanding, minor manipulation of Vas deferens and vessels during dissection of sac and closing it on the highest point^7^. Most pediatric surgeons incise the external oblique aponeurosis and by specifying the inner ring they release the cord^3^. Another group of pediatric surgeons use another method named Michelle banks. This technique is without incising external oblique aponeurosis, and hernia sac is closed at the outer ring outside of the canal^5^. It´s known that the main cause to hernia recurrence is an inadequate sac closure in upper area. According to literature, incising external oblique aponeurosis is most recommended. Other studies say that in children under two years the inguinal canal is too short to have separated inner and outer rings. It is recommended that all surgeries can be done without incising external oblique aponeurosis and distal to unopened ring^8^. 

Due to the high incidence of pediatric inguinal hernia, different surgical techniques and lack of an overall operation procedure selection agreement among pediatric surgeons, we intend to compare the results and complications between two conventional methods of pediatric inguinal herniotomy, with and without incising external oblique aponeurosis, in terms of recurrence and other complications.

## METHODS

This study was registered in Iranian Registry of Clinical Trial IRCT ID: IRCT2016041727446N1.

In this blinded randomized clinical trial, 800 children with indirect inguinal hernia candidate for herniotomy in the general surgery wards in Imam Khomeini and Abuzar Children’s Hospital, Iran, were evaluated from 2014 to 2015. The study was approved by Ethical Committee of Ahvaz Jundishapur University of Medical Sciences (Ref. No. IR.AJUMS.REC.1394.478) and all parents’ patients signed the consent form. 

Inclusion criteria included children with inguinal hernia. The exclusion ones, patients with hydrocele, undescending testis, underlying disease, sliding hernia and incarceration hernia. 

They were divided into two 400 patients groups. The first underwent herniotomy without incising external oblique aponeurosis and the second underwent herniotomy with incising external oblique aponeurosis and canal, and closing the sac in inner ring.

It was blinded study whereas patients were unaware of type of the surgery. Surgeon blinding was not possible due to the type of the study. Demographic and clinical variables studied included age (months), gender, hernia recurrence, ileoinguinal nerve damage (surgeon observation during surgery, as cut or trauma and crush), hematoma, seroma (accumulation of localized blood or operation diffused bruises), testicular ascent (testicles touched in the inguinal canal), hydrocele (scrotal fluid accumulation and scrotum enlargement without color changing), testicular atrophy (testicles different in size and being smaller than another one through the examination and ultrasound) and ischemic orchitis (painful, rigid and large testicle) were evaluated. Complications were considered as hematoma, seroma, hydrocele, testicular ascent, testicular atrophy, ischemic orchitis confirmed by ultrasound after surgeon´s diagnosis.

The main outcome of this study was to evaluate the hernia recurrence rate in each of two surgical procedures of pediatric herniotomy. Hernia recurrences at one year after surgery were evaluated. Secondary outcomes included comparison of other herniotomy complications one year after surgery in the two groups.

### Statistical analysis

Was performed using SPSS software Statistics for Windows, Version 22.0.(Chicago: SPSS Inc, Chicago, Illinois, USA). Chi-Square test was used to compare nominal variables. The odds-ratio was used in order to evaluate complications with or without incising external oblique aponeurosis. p<0.05 was considered significant. 

## RESULTS

Eight hundred children with inguinal hernia were analyzed. Four hundred were submitted to herniotomy without incising external oblique aponeurosis and 400 with. The complication incidence rates after one year of herniotomy based on age groups are shown in Table 1. Most groups requiring herniotomy were three months to two years old, and in total less than five years old.

**TABLE 1:** The study characteristics and complications rates one year after surgery based on age groups

Variables			Age Event / Total (Prevalence)				p
			6 to 12 years old	3 to 5 years old	3 months to 2 years old	Less than 3 months	
Type of surgery	With incising external oblique aponeurosis		86/400 (%21.5)	186/400 (% 46.5)	98/400 (% 24.5)	30/400(% 7.5)	0.97
	Without incising external oblique aponeurosis		90/400 (% 22.5)	180/400 (% 45.0)	100/400 (% 25.0)	30/400 (% 7.5)	
Hernia recurrence			0/176 (% 0)	(% 1.1) 4/366	4/198 (% 2)	0/60 (% 0)	0.267
Surgical hematoma			5/176 (% 2.8)	(% 2.5) 9/366	3/198 (% 1.5)	1/60 (% 1.7)	0.86
Inguinal nerves damages			4/176 (% 2.3)	6/366 (% 1.6)	1/198 (% 0.5)	0/60 (% 0)	0.44
Abdominal viscera damage (appendix)			2/176 (% 1.1)	0/366 (% 0)	0/198 (% 0)	0/60 (% 0)	0.124
Hydrocele following surgery			14/144 (% 9.7)	30/290 (% 10.3)	(% 13.8) 24/174	8/46 (% 17.4)	0.356
Ascending testis			3/144 (% 2.1)	3/290 (% 1)	1/174 (% 0.6)	0/46 (% 0)	0.64
Testicular atrophy			3/176 (% 1.7)	3/366 (% 0.8%)	1/198 (% 0.5%)	(% 0) 0/60	0.52
Ischemic orchitis			2/144 (% 1.4)	4/290 (% 1.4)	0/174 (% 0)	0/ (% 0)	0.449
Vas deferens damage			2/144 (% 1.4)	2/290 (% 0.7)	0/174 (% 0)	0/46 0)	0.456

The complication incidence rates after one year of herniotomy, based on the type of surgery, are shown in Table 2. In relation to the different groups - without and with incising external oblique aponeurosis - the results were, respectively: a) hernia recurrence, n=4 (1%) vs. n=4 (1%); b) hematoma, n=5 (1.3%) vs. n=13 (3.3%); c) nerve damage, n=2 (0.5%) vs. n=9 (2.3%); d) abdominal viscera damage, n=0 (0%) vs. n=2 (0.5%, p=0.499 no significant); e) hydrocele, n=24 (7.4%) vs. n=52 (15.9%); f) testicular size change, n=1 (0.3%) vs. n=6 (1.8%); g) ischemic orchitis, n=2 (0.6%) vs. n=4 (1.2%); h) vas deferens damage, n=2 (0.6%) vs. n=2 (0.6%) 

Odds ratio of complications of each technique is presented in Table 3. Hydrocele odds ratio of 2.371 in with incising external oblique aponeurosis group was similar to the group without (OR=2.371). This difference was statistically significant (p=0.001).

**TABLE 2:** Incidence rates of complications one year after surgery based on herniotomy type (with and without incising external oblique aponeurosis)

Variables	Type of surgery Event / Total (Prevalence)		p
	CWith incising external oblique aponeurosis	Without incising external oblique aponeurosis	
Hernia recurrence	4/400 (% 1)	4/400 (% 1)	1.000
Surgical hematoma	13/400 (% 3.3)	5/400 (% 1.25)	0.056
Inguinal nerves damages	9/400 (% 2.25)	2/400 (% 0.5)	0.064
Abdominal viscera damage (appendix)	2/400 (% 0.5)	0/400 (% 0)	0.490
Hydrocele following surgery	52/328 (% 15.9)	24/326 (% 7.36)	0.001
Testicular ascent	6/328 (% 18.3)	1/326 (% 0.3)	0.123
Testicular atrophy	1/400 (% 0.3)	6/400 (% 1.5)	0.058
Ischemic orchitis	4/328 (% 1.22/)	2/326 (% 0.61)	0.686
Vas defferens damage	2/328 (% 0.60)	6 (% 0.61)	1.000

**TABLE 3:** Complications odds ratio after herniotomy between the two groups, without and with incising external oblique aponeurosis

	Odds ratio	95% confidence interval	p
Hernia recurrence	1	0.248 - 4.026	1.000
Surgical hematoma	2.654	0.937 - 7.515	0.066
Inguinal nerves damages	4.581	0.983 - 21.335	0.053
Abdominal viscera damage (appendix)	0	0 - 0	0.99
Hydrocele following surgery	2.371	1.423 - 3.95	0.001
Testicular ascent	6.056	0.725 - 50.584	0.096
Testicular atrophy	0.165	0.02 - 1.373	0.096
Ischemic orchitis	2	0.364 - 10.99	0.425
Vas deferens damage	0.994	0.139 - 7.09	0.995

## DISCUSSION

Inguinal hernia is common disease in children^8^. Its repair complication rates in children have been reported less than 2%^5^. The most important factors in reducing the complications are included surgeon training, surgeon experience and also less manipulation.

Hematoma and scrotal swelling incidence are common when inguinoescrotal sac is large, and generally disappears about one month after surgery. Testicular atrophy in hernia repair occurs about 1% routinely.

In our previous study, recurrence rate was 2.2%^1^; Hughes et al reported it being 2.7%^2^, very similar to this one.

The most important difference between the two techniques in this study, was hydrocele incidence after surgery, being without incision group with 15.9% vs. 7.36% with incision. 

## CONCLUSION

Hernia recurrence and other postoperative complications were comparable between the two groups. Therefore, herniotomy without incising oblique aponeurosis can be appropriate replacement choice to herniotomy with incising oblique aponeurosis. Children with inguinal herniotomy can be benefit from herniotomy without incising oblique aponeurosis instead of more interventional traditional method.
